# Endovascular management of asymptomatic large descending thoracic aortic aneurysm long after coarctation of aorta repair: a case report

**DOI:** 10.1186/s13019-026-04102-z

**Published:** 2026-05-08

**Authors:** Prabhnoor Nagra, Matthew Neely, Arunabh Sinha, Garrett Wolfram, Joel Corvera, Blair MacPhail, Raed Abdulkareem, Raghunandan Motaganahalli

**Affiliations:** 1 Indiana University School of Medicine, Indianapolis, IN USA; 2 Goshen Health, Goshen, IN USA

**Keywords:** Thoracic aortic aneurysm, Coarctation of the aorta, TEVAR, Carotid–carotid bypass, Congenital heart disease, Endovascular repair, Late complication, Aortic arch surgery

## Abstract

**Background:**

Coarctation of the aorta represents 6–8% of congenital heart defects. Surgical repair is often done in infancy, but late complications such as aneurysm formation may be present decades later. Late aneurysm after childhood coarctation repair remains a recognized complication, but surveillance lapses and complex arch branch involvement can delay detection and complicate repair planning. We present a case of a large descending thoracic aortic aneurysm found incidentally over 60 years after initial coarctation repair, a latency period exceeding many reported cases [5].

**Case presentation:**

A 62-year-old woman with a history of coarctation repairs at 5 months of age and at 10 years of age presented asymptomatically for echocardiogram evaluation of a cardiac murmur. Computed tomography angiography (CTA) revealed a 7.3 cm saccular aneurysm (length 8.2 cm, width 6.5 cm, depth 7.3 cm) of the distal aortic arch and proximal descending aorta, with the left subclavian artery previously ligated and the left carotid artery originating from the aneurysm sac. She underwent a hybrid repair performed in a single setting: right common carotid-to-left common carotid bypass via retro-pharyngeal tunneling followed by thoracic endovascular aortic repair (TEVAR) with overlapping stent grafts. Angiography confirmed complete exclusion of the aneurysm without endoleak. Follow-up revealed no complications, and the patient remained asymptomatic.

**Conclusions:**

This case underscores the importance of lifelong surveillance in patients with repaired congenital heart disease, even in the absence of symptoms. Evolution of technology with endovascular repair has provided less invasive options for management of complex aneurysms resulting after repair of aortic coarctation. This case report demonstrates surveillance gaps after repair of thoracic aortic coarctation.

## Background

Coarctation of the aorta accounts for approximately 6–8% of all congenital heart defects, characterized by narrowing of the thoracic aorta. Surgical repair is typically performed in infancy or childhood. Despite successful early outcomes, patients remain at risk for long-term complications, including recurrent coarctation (34%), hypertension, and aneurysm formation at or near the repair site (20%) [1], with recent studies reporting up to 50% incidence after patch aortoplasty [2, 3]. Thoracic endovascular aortic repair (TEVAR) has become a preferred approach in selected patients with favorable anatomy, particularly in older, asymptomatic individuals [4, 5]. Indications for TEVAR include aneurysms > 5.5 cm or rapidly expanding, with contraindications such as connective tissue disorders or unfavorable access vessels [5, 6]. Although post-coarctation aneurysms and hybrid repair strategies have already been described in literature, late-presenting cases with branch vessel involvement raise practical planning questions regarding revascularization strategy, landing zone selection, and alternatives such as open redo repair or total endovascular arch repair. This case is an educational example emphasizing importance of surveillance protocol and contemporary management decision-making rather than technical novelty. We present a case of a very large 7.3 cm thoracic aortic aneurysm discovered incidentally in an adult female more than six decades after childhood coarctation repair.

## Case presentation

A 62-year-old woman with a history of aortic coarctation repair at 5 months and again at 10 years of age was referred for evaluation following detection of a cardiac murmur on physical exam. Original operative records from childhood repairs are unavailable due to the remote timeframe, however intraoperative and imaging findings of a ligated left subclavian artery used as a patch are consistent with subclavian flap aortoplasty, a technique commonly performed in infants during the 1960–1970 s era of the initial repair [1, 2]. The redo repair at age 10 was presumably for recurrent coarctation, a known late complication following such techniques, particularly in young patients [3, 7]. Records of the age-10 evaluation, including echocardiography findings, were unavailable. She reported no symptoms but did note life-long blood pressure discrepancies between her arms. Pre-procedure blood pressures included measurements of 136/82 and 137/88 mmHg in her right arm with 30 mm Hg gradient in the left arm pressure. Lower limb and exercise measurements were not documented. Medical history included Raynaud’s phenomenon and was not felt to be contributory to aneurysm development or operative decision-making. She had no prior cardiovascular symptoms such as chest pain, dyspnea, or hoarseness. She had a cardiac murmur during the pre-op examination. Home medications included losartan 50 mg daily, metoprolol succinate 50 mg daily, aspirin 81 mg daily, atorvastatin 20 mg.

**Fig. 1:**
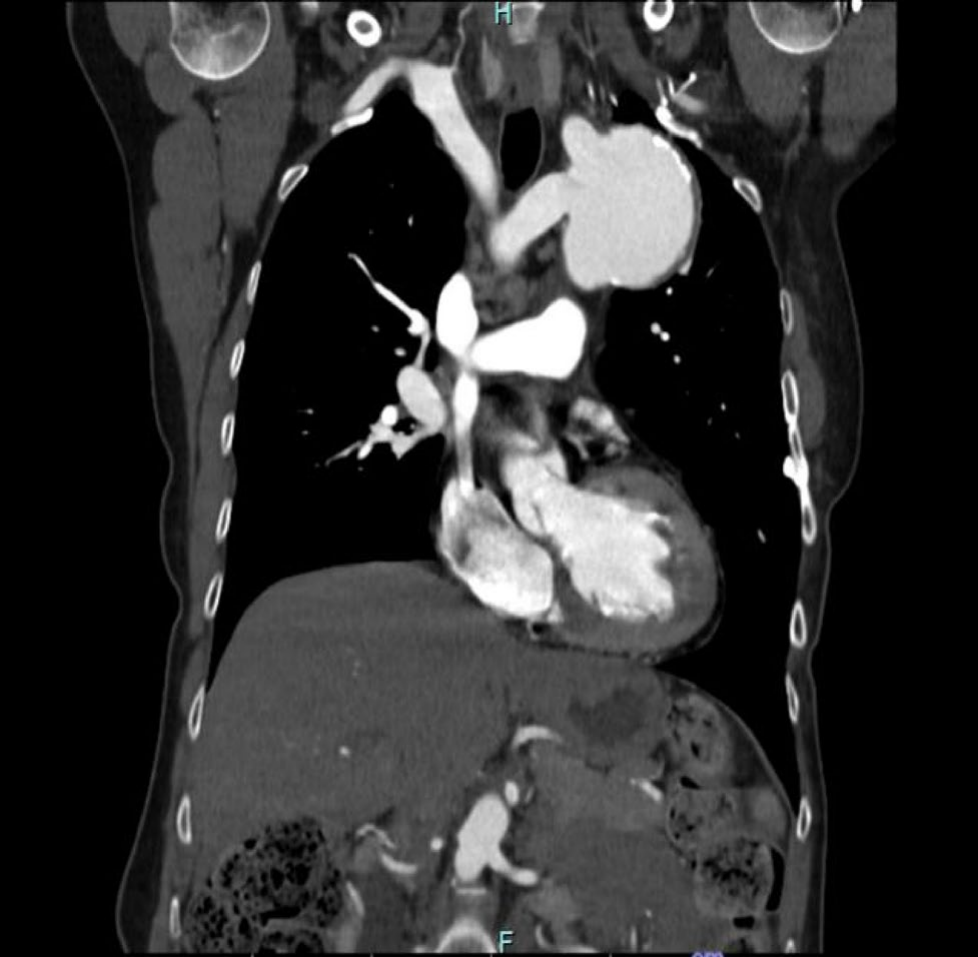
Computed tomography angiography (CTA) showing a large 7.3 cm saccular aneurysm of the distal thoracic aortic arch and proximal descending aorta in the coronal plane

**Fig. 2:**
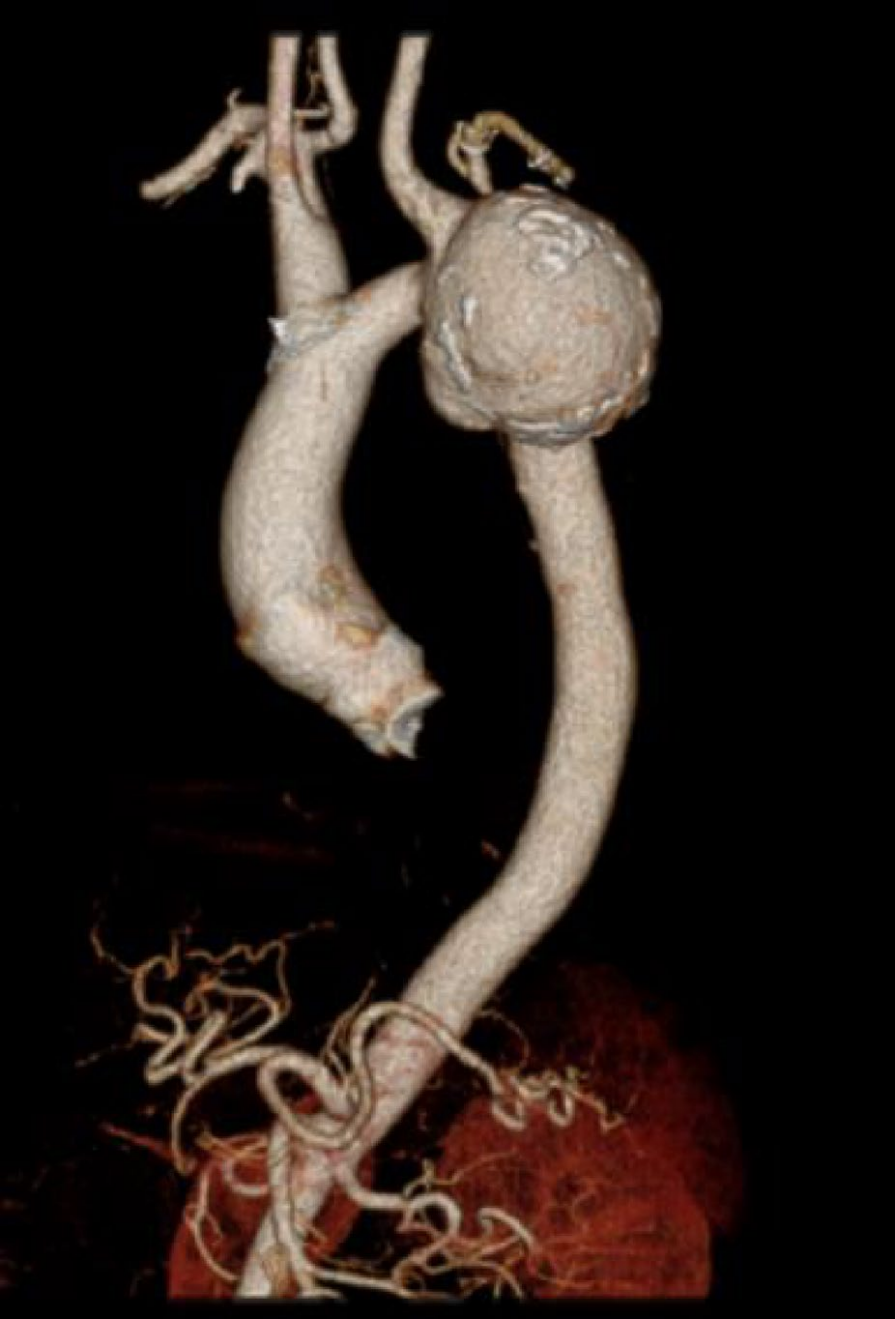
Three-dimensional CTA reconstruction demonstrates the aneurysm with the left carotid artery originating from the aneurysmal sac

Transthoracic and transesophageal echocardiography revealed a bicuspid aortic valve with thickened aortic valve leaflets and trace insufficiency (no stenosis, valve area 1.14 cm²), ascending aorta 3.4 cm, and proximal descending aortic aneurysm (~ 6.8 cm at left carotid origin on TEE, though not accurately measurable); left subclavian artery occlusion; normal ventricular function; no arch hypoplasia or collaterals. No pre-procedure electrocardiogram was documented. Coronary artery disease was excluded based on echocardiography and computed tomography angiography (CTA) without evidence of obstruction (no stress testing or catheterization performed). Carotid duplex showed no significant stenosis. CTA demonstrated a 7.3 cm saccular aneurysm involving the distal thoracic aortic arch and proximal descending aorta (Fig. 1). The left subclavian artery had been previously ligated during her initial repair and was used as a patch. The left carotid artery was found to originate from the aneurysmal sac, as shown on three-dimensional CTA reconstruction (Fig. 2). This configuration limited an adequate proximal landing zone and necessitated branch revascularization to preserve cerebral perfusion while achieving durable aneurysm exclusion.

**Fig. 3:**
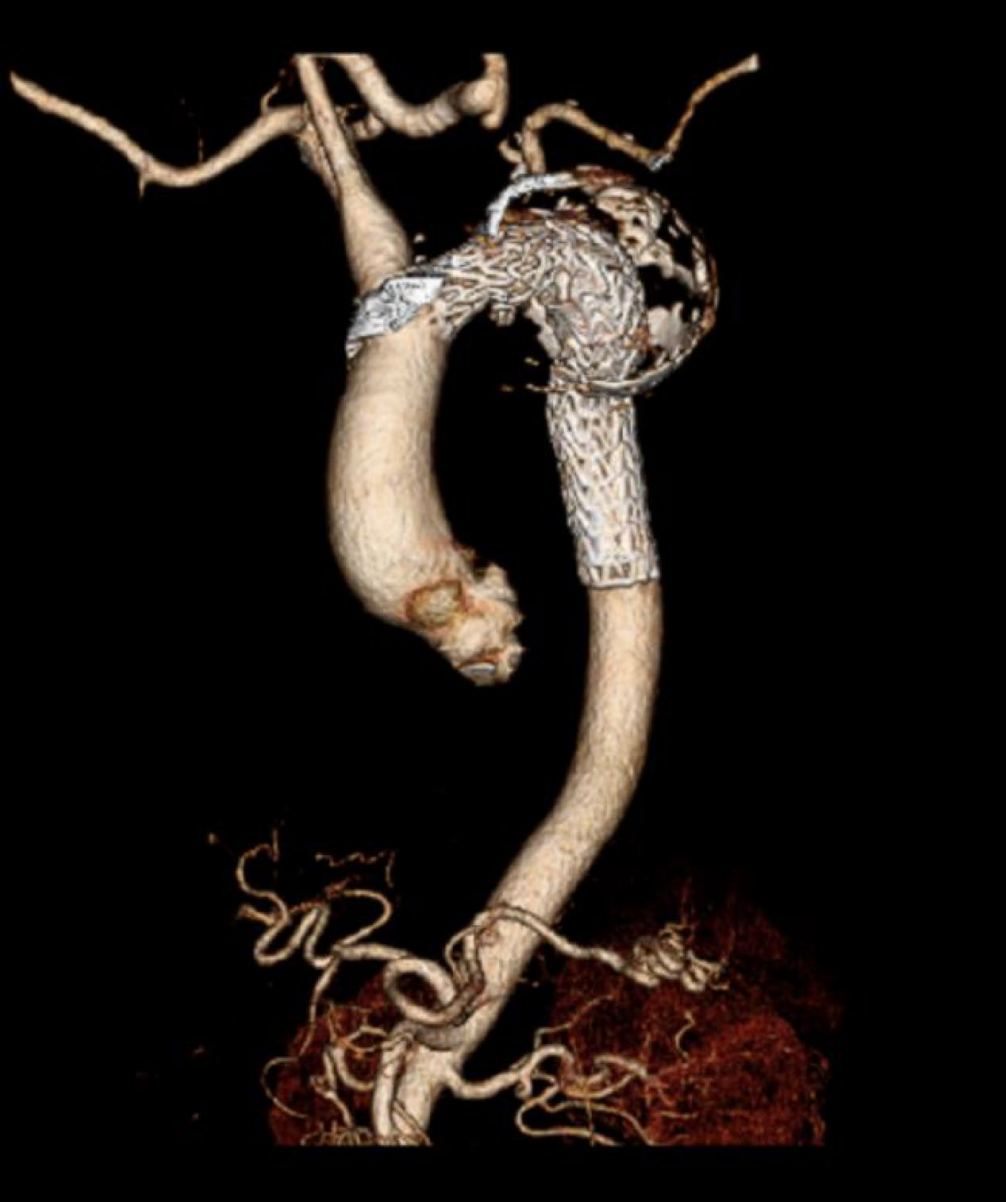
Three-dimensional CTA reconstruction showing repair and exclusion of the aneurysm with TEVAR

Due to the aneurysm’s large size (> 5.5 cm) and risk of rupture, a hybrid repair was planned and completed in a single setting. First, a right common carotid -to-left common carotid artery bypass was performed using an internally supported 8 mm polytetrafluoroethylene (PTFE) graft placed through a retro-pharyngeal tunnel, followed by ligation of the left carotid artery below the anastomosis. PTFE was selected due to conduit availability and caliber match; carotid to carotid transposition was considered but not pursued due to anatomic constraints and operative exposure considerations. The patient was maintained on antiplatelet therapy postoperatively. Next, TEVAR was performed via bilateral femoral access. A 28 mm × 15 cm Gore CTAG (WL Gore, Flagstaff, AZ) stent graft was deployed just distal to the innominate artery. Due to the large, cavernous sac, stent graft retracted requiring two additional grafts of 28 mm X15 cm and a 28 mm X 10 cm to provide adequate overlap between the stent grafts while extending the proximal seal in Zone 1. Final angiography showed successful exclusion of the aneurysm with no evidence of endoleak (Fig. 3).

At follow-up, the patient reported occasional elevated heart rates (100–120 bpm) but denied chest pain, palpitations, or respiratory symptoms. She remained hemodynamically stable and had no post-procedural complications. Day one post-TEVAR blood pressure was 103/52 mmHg (no limb/exercise differences documented), renal function was stable. At one-year follow-up patient had satisfactory repair without graft migration or endoleak. She remained asymptomatic. Surveillance was planned annually in alignment with current aortic disease guideline recommendations.

## Discussion

This case highlights several important principles in the long-term management of patients with repaired congenital heart disease. Despite successful childhood repair, our patient developed a massive thoracic aortic aneurysm that remained undetected until her sixth decade, a latency exceeding most reported cases and underscoring the need for vigilant lifelong surveillance with imaging (CTA/MRA) at least every 5 years [2, 5]. The patch-based and subclavian flap repair techniques are typically associated with altered flow dynamics and progressive aortic wall degeneration, predisposing to late aneurysm formation. Concomitant bicuspid aortic valve disease further increases long-term aortopathy risk [5]. These mechanisms explain the potential for aneurysm development decades after apparently successful repair.

This patient did not receive the recommended follow-ups despite a history of coarctation and a long-standing cardiac murmur, as she reports not being adequately informed of her increased risk of thoracic aortic aneurysm (TAA) and its potential presentation. The presence of the left subclavian artery patch increased her risk for aneurysm formation, a fact that may not have been recognized or communicated to the patient over the years [8]. Lack of awareness, lack of education pertaining to lifelong surveillance likely contributed to delayed detection [2, 9]. This case truly underscores the importance of structured congenital to adult care transition and adherence of surveillance; absence of symptoms does not protect one from progressive degeneration of aneurysms.

TEVAR is a valuable minimally invasive treatment for descending thoracic aneurysms, offering a favorable alternative to open surgery in select patients. An open redo repair carries increased risks related to re-entry, bleeding, recurrent laryngeal nerve injury, and spinal cord ischemia [10]. A hybrid approach as in this case provides a durable novel solution. TEVAR using branched or fenestrated devices is an emerging alternative but remains dependent on patient anatomy, device availability, and long-term durability considerations [11]. However, anatomical complexities, such as aneurysmal involvement of major branches, may require adjunctive procedures like carotid-to-carotid bypass. In our case, the decision to perform both interventions in one setting minimized the patient’s procedural risk and hospitalization time. Endovascular repair is an attractive option considering the risks of open redo surgery such as increased risk of bleeding, paraplegia, recurrent nerve palsy, and difficult exploration resulting in higher morbidity in reinterventions [5, 6]. Hybrid approaches, as used here, provide safe outcomes in complex cases, with low mortality in recent series [3, 12]. The contribution of this case is educational as it demonstrates late post-coarctation aneurysm progression beyond 60 years, progressive proximal branch vessel involvement arising from the aneurysm sac, and the evolution of endovascular technology, hybrid procedures to provide a durable treatment option in this complex pathology.

## Conclusion

This case demonstrates the critical importance of lifelong surveillance in patients with repaired congenital heart and aortic pathology. Patient education specifically with need for lifelong surveillance is paramount to preventing life threatening complications. Evolution of Endovascular repair, combined with arch de-branching procedures, can provide a safe and effective solution in complex anatomical cases, particularly where redo procedures carry significant complications.

## Data Availability

No datasets were generated or analysed during the current study.

## Abbreviations

CTA: Computed Tomography Angiography
PTFE: Polytetrafluoroethylene
TAA: Thoracic Aortic Aneurysm
TEVAR: Thoracic Endovascular Aortic Repair
